# MDPET: A Unified Motion Correction and Denoising Adversarial Network for Low-Dose Gated PET

**DOI:** 10.1109/TMI.2021.3076191

**Published:** 2021-10-27

**Authors:** Bo Zhou, Yu-Jung Tsai, Xiongchao Chen, James S. Duncan, Chi Liu

**Affiliations:** Department of Biomedical Engineering, Yale University, New Haven, CT 06511 USA; Department of Radiology and Biomedical Imaging, Yale University, New Haven, CT 06511 USA.; Department of Biomedical Engineering, Yale University, New Haven, CT 06511 USA; Department of Biomedical Engineering and the Department of Radiology and Biomedical Imaging, Yale University, New Haven, CT 06511 USA

**Keywords:** Low-dose gated PET, denoising, motion estimation, motion correction, unified network, deep learning

## Abstract

In positron emission tomography (PET), gating is commonly utilized to reduce respiratory motion blurring and to facilitate motion correction methods. In application where low-dose gated PET is useful, reducing injection dose causes increased noise levels in gated images that could corrupt motion estimation and subsequent corrections, leading to inferior image quality. To address these issues, we propose MDPET, a unified motion correction and denoising adversarial network for generating motion-compensated low-noise images from low-dose gated PET data. Specifically, we proposed a Temporal Siamese Pyramid Network (TSP-Net) with basic units made up of 1.) Siamese Pyramid Network (SP-Net), and 2.) a recurrent layer for motion estimation among the gates. The denoising network is unified with our motion estimation network to simultaneously correct the motion and predict a motion-compensated denoised PET reconstruction. The experimental results on human data demonstrated that our MDPET can generate accurate motion estimation directly from low-dose gated images and produce high-quality motion-compensated low-noise reconstructions. Comparative studies with previous methods also show that our MDPET is able to generate superior motion estimation and denoising performance. Our code is available at https://github.com/bbbbbbzhou/MDPET.

## I. Introduction

Positron emission tomography (PET) is a commonly used functional imaging modality with wide applications in oncology, cardiology, neurology, and biomedical research. PET scans require injection of a small amount of radioactive tracer to patients, introducing radiation exposure to both patients and healthcare providers. By reducing the administered injection dose, low-dose PET is of-great-interests according to the As Low As Reasonably Achievable concept (ALARA) [1], in particular for applications of serial PET scans to measure response to therapy. Since the data acquisition typically takes 10 to 20 minutes, the patient’s respiratory motion in the thorax and upper abdomen areas inevitably introduces blurring in the reconstructed images, affecting subsequent diagnosis and treatments [2]. Respiratory gating facilitated by external motion monitoring devices, such as Anzai [3], is typically used to provide gated images with reduced respiratory motion effect. The gated image that shows minimum motion effects is then used for clinical interpretation. However, the interpretation can still be hampered by the increased image noise level as each gated image is generated by only a fraction of all detected events. To tackle the issue, previous works proposed approaches involving an initial image reconstruction for each gate followed by an image registration for motion estimation among different gates. The motion vectors derived from the image registration were then utilized to average transformed images or incorporated into a final reconstruction to generate a motion compensated image with all events. In addition to using the conventional non-rigid image registration algorithms [4]–[7], deep learning based methods were explored recently as well [8], [9]. However, the noisy gated images could lead to inaccurate motion estimation and alignment errors. In applications of low-dose gated PET, this makes extending the previously mentioned approaches for motion estimation/correction challenging because the noise level is further increased in each gated images. The highly noisy gated image could lead to non-ideal motion estimation results by previous methods, and could subsequently degrade the final motion-compensated reconstructions. Moreover, in low-dose gated PET, denoising methods should also be applied to the final motion-compensated image reconstructed with all events because there are limited events from low-dose data.

Previous works on denoising low-dose PET can be summarized into two categories: conventional image post-processing [10]–[12] and deep learning based methods [13]–[22]. Conventional image post-processing techniques, such as Gaussian filtering, are standard techniques in practice, but have challenges to preserve local structures. Non-local mean filter [10] and block-matching 4D filter [11] were proposed to denoise low-dose PET while better preserving the structural information. Although these conventional image post-processing methods may substantially improve the image quality, over-smoothing is often observed in ultra-low-dose data. Recently, deep learning techniques have achieved promising performance in medical imaging applications, such as reconstruction [23]–[27], segmentation [28]–[30], registration [31] and denoising [32]. As the statistical characteristics of noise in medical imaging is complex and hard to model, deep learning models can learn the highly non-linear relationship from data and recover the original signal from noise. For deep learning based low-dose PET denoising, previous works can be further divided into two categories. The first category only uses the low-dose PET data as input. Kaplan and Zhu [16] proposed using a GAN [33] with UNet [28] as generator to predict standard-dose PET images from low-dose PET images. Similarly, Wang et el. [14] proposed using a 3D-conditional-GAN [34] also with UNet as generator to translate low-dose PET images to standard-dose PET images. In addition to GAN, Ouyang *et el*. [20] further improves the denoising performance by incorporating patient specific diagnosis information. Zhou *et el*. [19] and Gong *et el*. [18] found incorporating Wasserstein GAN [35] can also achieve promising low-dose PET denoising performance. Furthermore, Hu *et el*. [17] proposed a DPIR network that directly predicts the standard-dose PET image from low-dose PET sinogram data. The second category uses the low-dose PET images and MR/CT images as input. Xiang *et el*. [13] proposed a deep auto-context CNN that takes low-dose PET image and T1 MR image as input for prediction of standard-dose PET image. Similarly, Chen *et el*. [21] proposed to input low-dose PET images along with multi-contrast MR images into a UNet [28] for ultra-low-dose PET denoising. Cui *et el*. [36] suggested to use a UNet to iteratively predict the denoised PET from the CT image. Comparing to conventional PET denoising methods, all these deep learning based methods achieved superior denoising performance on static low-dose PET.

**Fig. 1.**
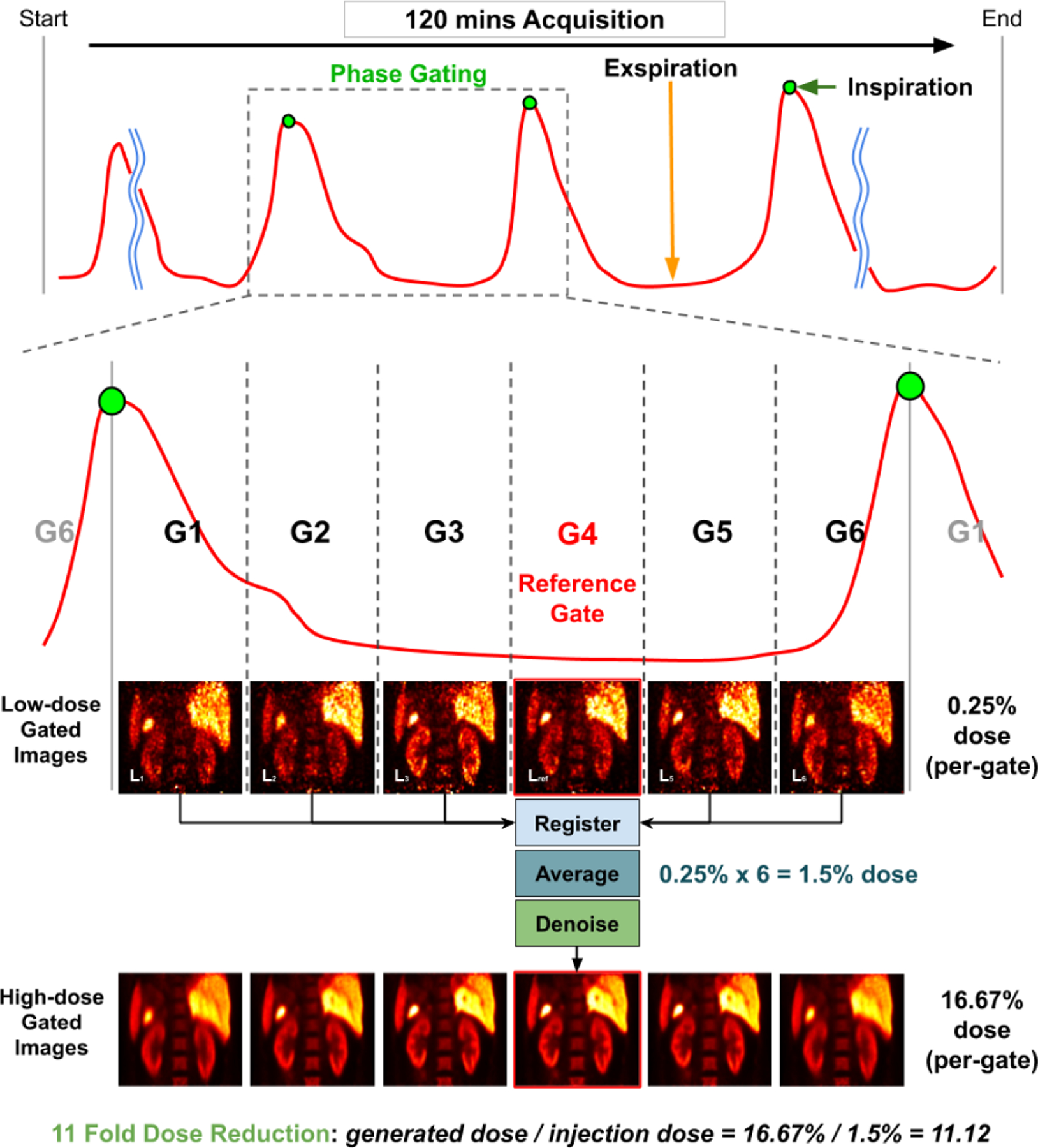
Illustration of phase gated PET and the proposed method. The Anzai signal (red curve) can guide the assignment of the detected events to different respiratory phases and generate 6 gated images. End-expiration gate with the least intra-gate motion (G4) is used as our reference gate. Our goal is to register all the low-dose gated images to the reference gate, averaging them, and denoise the averaged image to generate a high-dose gated image at the reference gate with the least intra-gate motion.

**Fig. 2.**
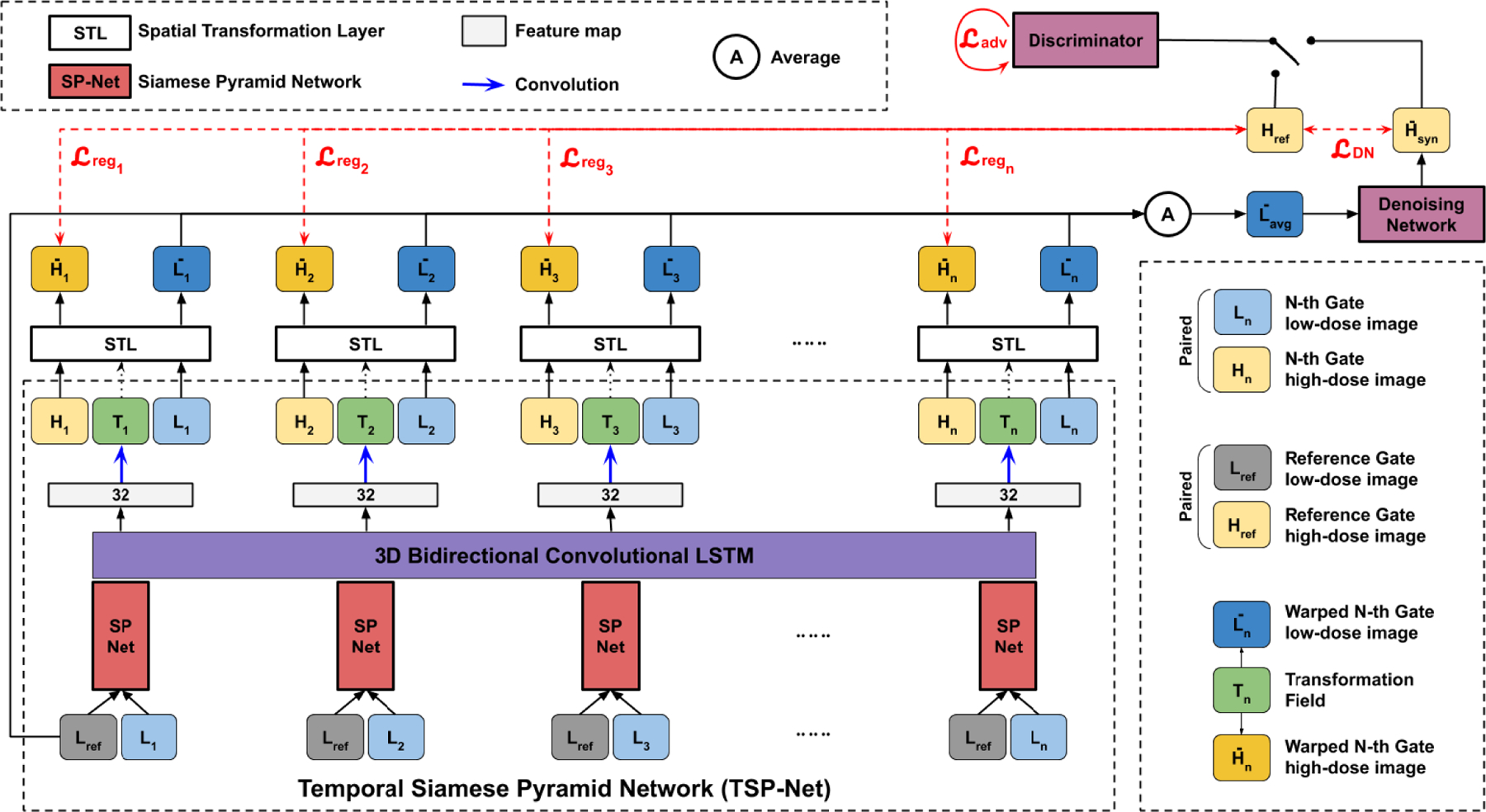
The overall structure of our unified motion correction and denoising network (MDPET). The reference gate low-dose image *L*_ref_ and N-th gate low-dose images *L_n_* are fed into each Siamese Pyramid Network (SP-Net) within our Temporal Siamese Pyramid Network (TSP-Net). The predicted transformation fields *T_n_* simultaneously transform the paired *L_n_* and *H_n_*. The transformed low-dose gated image ${\hat{L}}_{n}$ are averaged and subsequently fed into the denoising network for denoising. Our MDPET is trained in a unified fashion with registration loss $L_{\text{reg}}$, denoising loss $L_{DN}$, and adversarial loss $L_{\text{adv}}$ combined.

**Fig. 3.**
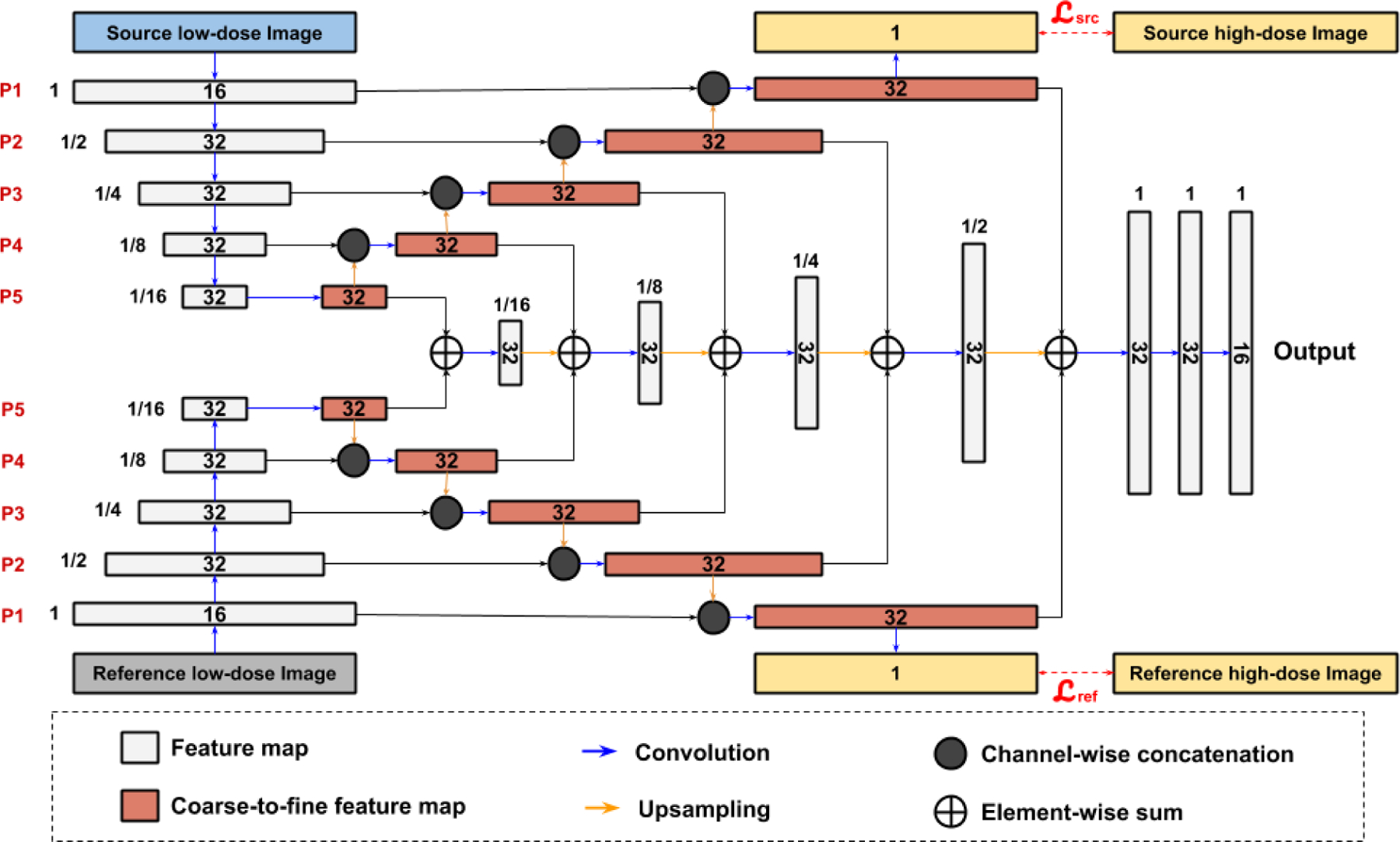
Design of our Siamese Pyramid Network (SP-Net). 5 levels of pyramid features are generated from the reference low-dose gated image *L_ref_* and the source low-dose image *L_n_*. Generation of pyramid features are supervised by the reference high-dose image *H_ref_* and the source high-dose image *H_n_*. The pyramid features are fused and decoded to generate the transformation features. The number of feature channel is denoted inside the feature map. The spatial resolution of each feature map with respect to the input image is printed next to the feature map.

However, none of the above mentioned studies addressed motion estimation and denoising in low-dose respiratory gated PET. Recently, our group proposed a Siamese Adversarial Network (SAN) to estimate the motion between pairs of low-dose gated images by first denoising the low-dose gated images and estimating the motion based on them [37]. One limitation of this approach is that the motion estimation network only considers pairs of gated images for registration and relies on high-quality denoised images of each gates, while disregarding the temporal information over the gated images. The temporal information containing respiratory motion patterns may be potentially helpful for motion estimation tasks. Therefore, it is desirable to develop a motion estimation algorithm that does not rely on denoised low-dose gated images and can directly estimate the motion from original low-dose gated images, while incorporating the temporal information among gates. With accurate motion estimation from low-dose gated images, we can register the low-dose gated images to a reference low-dose gated images and average all the aligned low-dose gated images to generate a motion-compensated PET image with preliminary denoising. This image can be fed into another deep network for further denoising. The general pipeline of the idea is illustrated in Figure 1. In this work, we design a unified motion correction and denoising adversarial network for low-dose gated PET, called MDPET. As illustrated in Figure 2, our MDPET is a unified network consisting of a Temporal Siamese Pyramid motion estimation network (TSP-Net), a denoising network, and a discriminator. Specifically, our TSP-Net consists of multiple shared-weights Siamese Pyramid Networks (SP-Net) and a bi-directional LSTM (Figure 3). Each SP-Net predicts the transformation field between the source gated image and the reference gated image by utilizing the coarse-to-fine pyramid features from pairs of low-dose gated images. After registering all the source low-dose gated images with the reference low-dose gated image via Spatial Transformation Layers (STL) [38], the average image is fed into the denoising network for generation of our final motion-compensated denoised PET image. The network structure and training details are described in the following sections. The experimental results on human data demonstrate that our MDPET can accurately estimate the motion from low-dose gated images and generate high-quality motion-compensated PET images.

## II. Problem Formulation

As illustrated in Figure 1, assuming a phase gated PET scan generates 6 gates, we denote high-dose gated images and low-dose gated images as *H*_*n*_, $L_{n}\in {\mathbb{R}}^{h\times w\times d}$ with gate index of *n* ∈ {1, 2, 3, 4, 5, 6} and image size of *h* × *w* × *d*. Here, typical end-expiration gate 4 with the least intra-gate motion is used as our reference gate, and we denote *H*_*ref*_ = *H*_4_ and *L*_*ref*_ = *L*_4_, respectively.

First, our goal is to accurately estimate a set of transformation fields *T*_*n*_ between *L*_*ref*_ and *L*_*n*_ with *n* ∈ {1, 2, 3, 5, 6}. Denoting our motion estimation model as $P_{T\;S\;P}$ parameterized by *θ*_*TSP*_, the transformation fields can be described as:

(1) $$T_{1},\cdots ,T_{n}=P_{T\;S\;P}\left(L_{1},\cdots ,L_{n};L_{ref},\theta_{T\;S\;P}\right)$$

Each transformation field *T*_*n*_ is used to deform the low-dose gated image *L*_*n*_ to generate an average image *L*_*avg*_:

(2) $$L_{avg}=\frac{1}{N}\left(L_{ref}+\underset{n\neq ref}{\sum }T_{n}\circ L_{n}\right)$$

where *N* = 6 for 6 gates in our experiments. Then, our goal is to denoise the motion-compensated low-dose averaged image and generate a high-quality final PET image. Denoting our denoising model as $P_{DN}$ parameterized by *θ*_*DN*_, the denoised motion compensated average low-dose image is given by:

(3) $$H_{syn}=P_{DN}\left(L_{avg};\theta_{DN}\right)$$

Our customized motion estimation model $P_{T\;S\;P}$, denoising model $P_{DN}$, and the unified training strategy are discussed in details in the following section.

## III. Methods

### A. Unified Motion Estimation and Denoising Adversarial Network

The general pipeline of our unified motion estimation and denoising network (MDPET) is illustrated in Figure 2. Our MDPET consists of a motion estimation module and a denoising module. The two modules are unified and trained in an end-to-end fashion.

#### 1) Motion Estimation Network:

We build a Temporal Siamese Pyramid Network (TSP-Net) consisting of basic units of Siamese Pyramid Network (SP-Net) and a Bidirectional Convolutional Long Short Term Memory (BiConvLSTM) [39]. Each SP-Net is responsible for generating features for predicting the transformation between each source low-dose gated image *L*_*n*_ and the reference low-dose gated image *L*_*ref*_ with all SP-Nets share the same network parameters. Details of our SP-Net are provided in Figure 3. In general, our SP-Net has two input branches for generating coarse-to-fine pyramid features of the reference low-dose gated image *L*_*ref*_ and the source low-dose gated images *L*_*n*_ separately. Then, the coarse-to-fine pyramid features are fed into our decoder for estimating transformation, similar to the image pyramid used in traditional image registration methods [40]. More specifically, we use two 3D UNet in each SP-Net for generating 5 levels of pyramid features with goals of learning coarse-to-fine features and denoising the input images for robust feature representations. To achieve these goals, the finest decoded feature maps from the source low-dose image *L*_*n*_ and the reference low-dose image *L*_*re*_*f* are passed through two 1-channel 3D convolutional layers, and the outputs $\hat{H}$ are supervised by the high-dose gated images *H* with mean square error loss (MSE):

(4) $$L_{S\;P_{n}}=L_{ref}+L_{src_{n}}$$

(5) $$=\frac{1}{\left|H\right|}\underset{p}{\sum }{\left[H_{ref}(p)-{\hat{H}}_{ref}(p)\right]}^{2}$$

(6) $$+\frac{1}{\left|H\right|}\underset{p}{\sum }{\left[H_{src_{n}}(p)-{\hat{H}}_{src_{n}}(p)\right]}^{2}$$

where *p* denotes the voxel location in the images. |*H*| is the number of voxel in each image. *n* is the index of the gates. $L_{ref}$ and $L_{src_{n}}$ are the losses for reference gated image branch and source gated image branch, respectively. As illustrated in Figure 3, the pyramid feature maps from the UNet’s decoder successively recover the original high-dose signal from the low-dose signal, thus providing noise-reduced feature representations at different levels. Then, the coarse-to-fine pyramid features from the reference image and source image are successively fused together and decoded to generate features for predicting the transformation.

While each SP-Net generates features for predicting the transformation between the reference low-dose image and one of the source low-dose gated images, the adjacent and non-adjacent SP-Net’s features can provide additional non-local information, such as motion pattern in a full respiratory cycle, which can be potentially helpful for accurate motion estimation over low-dose gated images. Recurrent convolutional neural network, such as BiConvLSTM, is able to learn the feature pattern among correlated data samples over time. The cell state of BiConvLSTM allows temporal feature from adjacent or non-adjacent frames to be transferred along forward and backward temporal directions. Therefore, we concatenate a 3D BiConvLSTM to the output features of the SP-Nets to allow the temporal feature exchange from different gate’s motion estimation features (TSP-Net). The output features with 32 channels, as shown in Figure 2, are then fed into convolutional layers with 3 channels of output for predicting the transformation fields *T*_*n*_ over the gates.

For each gate, the spatial transformation layer [38] transforms both the high-dose gated image *H*_*n*_ and the low-dose gated image *L*_*n*_ with the predicted transformation field *T*_*n*_ from the TSP-Net. The loss function for supervising the motion estimation here can be written as:

(7) $$L_{reg}=\underset{n}{\sum }L_{reg_{n}}=\underset{n}{\sum }\left(L_{{\text{sim}}_{n}}+\lambda L_{{\text{smooth}}_{n}}\right)$$

with

(8) $$L_{{\text{sim}}_{n}}=\frac{1}{\left|H\right|}\underset{p}{\sum }{\left[H_{ref}(p)-{\bar{H}}_{n}(p)\right]}^{2}$$

(9) $$=\frac{1}{\left|H\right|}\underset{p}{\sum }{\left[H_{ref}(p)-\left[T_{n}\circ H_{n}\right](p)\right]}^{2}$$

(10) $$L_{{\text{smooth}}_{n}}=\underset{p}{\sum }{\left\|\nabla T_{n}(p)\right\|}^{2}$$

where *n* is the index of the gates. ${\bar{H}}_{n}$ is the transformed *H*_*n*_ with transformation field *T*_*n*_. $L_{{\text{sim}}_{n}}$ is the mean square error in image appearance, and $L_{{\text{smooth}}_{n}}$ is a deformation regularization that adopts a L2-norm of the gradient of the transformation field *T*_*n*_ with a weighing term of *λ*. As suggested in [9], we empirically set *λ* = 0.01 in our experiments.

#### 2) Unified With Denoising Network:

As mentioned above, the spatial transformation layer simultaneously transforms the low-dose gated image *L*_*n*_ with the predicted transformation field *T*_*n*_ from TSP-Net. Then, a motion-compensated low-dose gated image can be generated with:

(11) $${\bar{L}}_{avg}=\frac{1}{N}\left(L_{ref}+\underset{n\neq ref}{\sum }T_{n}\circ L_{n}\right).$$

where *N* = 6 for 6 gates setup in our experiments. While ${\bar{L}}_{avg}$ with 6 fold counts can significantly reduce the low-dose image’s noise, we further reduce the image noise by feeding ${\bar{L}}_{avg}$ to a denoising network. As UNet [28] has demonstrated outstanding performance in low-dose PET denoising [15], we adapt UNet as our denoising network in this work. However, our denoising network is not limited to UNet and can be substituted by other networks as well. The denoising loss can be formulated as:

(12) $$L_{DN}=\frac{1}{\left|H\right|}\underset{p}{\sum }{\left[H_{ref}(p)-{\bar{H}}_{syn}(p)\right]}^{2}$$

(13) $$=\frac{1}{\left|H\right|}\underset{p}{\sum }{\left[H_{ref}(p)-G\left({\bar{L}}_{avg}\right)(p)\right]}^{2}$$

where *G* is our denoising network and ${\bar{H}}_{syn}$ is the denoised image generated from ${\bar{L}}_{avg}$. Moreover, we incorporate a patch discriminator *D* for adversarial learning on the denoising output [34]. To achieve stable adversarial training, we used the LSGAN adversarial loss [41] that can be formulated as:

(14) $$L_{adv}={\left[D\left(H_{ref}\right)-1\right]}^{2}+[D{\left(G\left({\bar{L}}_{avg}\right)\right]}^{2}$$

Unifying the denoising network and the motion estimation network allows the denoising supervised gradient to back-propagate to the motion estimation network. As the denoising result relies on an accurate motion estimation over low-dose gated images and the alignment, the unified motion estimation and denoising adversarial network can be mutually beneficial. Therefore, the total loss for training our MDPET can be written as:

(15) $$L_{\text{tot}}=\lambda_{DN}L_{DN}+\lambda_{adv}L_{adv}+\lambda_{\text{reg}}L_{\text{reg}}+\lambda_{SP}\underset{n}{\sum }L_{SP_{n}}$$

where the weighting parameters are empirically set to *λ*_*DN*_ = 10, *λ*_*adv*_ = 1, *λ*_*reg*_ = 5, and *λ*_*SP*_ = 0.2 for a balance adversarial training.

### B. Evaluation on Human Data

We included 28 pancreas ^18^F-FPDTBZ [42] PET/CT studies. All PET data were obtained in list mode using the 4-ring Siemens Biograph mCT scanners located at the Yale PET Center. External respiratory motion was tracked using the AZ-733V respiratory gating system (Anzai Medical, Tokyo, Japan). The Anzai respiratory trace was recorded at 40 Hz for all subjects. The averaged dose administered to the patients is 9.13±1.37 mCi. Our patient dataset consists of 15 healthy patients and 13 Type-2 diabetic patients. All studies were approved by the Institutional Review Board and Radiation Safety Committee at Yale University. The total acquisition time was 120 mins for each study. We used phase gating to generate 6 gates for each study. To eliminate the mismatch between the attenuation correction (AC) map and the gated PET images, instead of using CT images to derive the AC-map, we utilized the maximum likelihood estimation of activity and attenuation (MLAA) [43] to generate AC-map for each gated volume to ensure phase-matched attenuation correction. The CT-derived AC-map was used as initial estimation for MLAA iterations. The high-dose images were reconstructed with 100% of the listmode data mimicking high radiation dose data with a large amount of tracer injection. Thus, each high-dose gated image was reconstructed with about 16.67% of the listmode data. The low-dose images were reconstructed with 1.5% of the listmode data with uniform sampling. Thus, each low-dose gated image was reconstructed with about 0.25% of the listmode data. Each dataset was reconstructed into a 400 × 400 × 109 volume with voxel size of 2.032 × 2.032 × 2.027 *mm*^3^ using ordered subset expectation maximization (OSEM) with 21 subsets and 1 iteration. The central 200 × 200 × 109 voxels were cropped to remove most voxels outside the human body contour. The resulted image was then resized to 96 × 96 × 96 voxels. The end expiration gate (typically Gate 4) was used as the reference gate since it shows minimum intra-gate motion.

We performed four-fold cross validation with each fold consisting of 7 studies. During each validation, 21 studies were used for training and 7 studies were used for testing. The evaluation was performed on all 28 studies with 6 gated images in each study. For motion estimation evaluation, the transformation fields estimated from low-dose gated images were used to transform the corresponding high-dose gated images, and then the Normalized Mean Absolute Error (NMAE) were computed between the reference high-dose gated image and the transformed high-dose gated images. For comparative study, we compared our motion estimation results against VoxelMorph (VM) [9], the previously proposed Siamese Adversarial Network (SAN) [37], and a non-deep learning based Non-Rigid B-spline Registration (NRB) implemented in BioImage Suite [40]. VM is a deep learning based registration framework that exhibits top-performance in a wide range of medical imaging applications. With NRB, we used normalized mutual information as the similarity metric and we set the parameter of control point spacing to be 15mm, same as the optimized parameters demonstrated in [7]. For denoising evaluation, we computed the Peak Signal-to-Noise Ratio (PSNR), Structural Similarity Index (SSIM), and NMAE between our final synthetic high-dose image and the reference high-dose gated image.

### C. Implementation Details

We implemented our method using Pytorch [44]. We used the ADAM optimizer [45] with a learning rate of 10^−4^. We set the batch size to 1 with each training batch consisting of gated images from one patient. We first pre-trained the TSP-Net by setting *λ*_*DN*_ = *λ*_*adv*_ = 0. Then, we pre-trained the denoising network using the predicted averaged images from our pre-trained TSP-Net and its denoising ground-truth. Finally, the pre-trained TSP-Net and denoising network were loaded into MDPET to train in an end-to-end fashion. Our model was trained on an NVIDIA Quadro RTX 8000 GPU for 200 epochs. To prevent overfitting, we also implemented ‘on-the-fly’ data augmentation for all the training steps. During training, we first resized the image to 106 × 106 × 106 and performed 96 × 96 × 96 random cropping, and then randomly rotated the images along the z-axis with angle between −30 to 30 degrees.

## IV. Results

### A. Motion Estimation

**Fig. 4.**
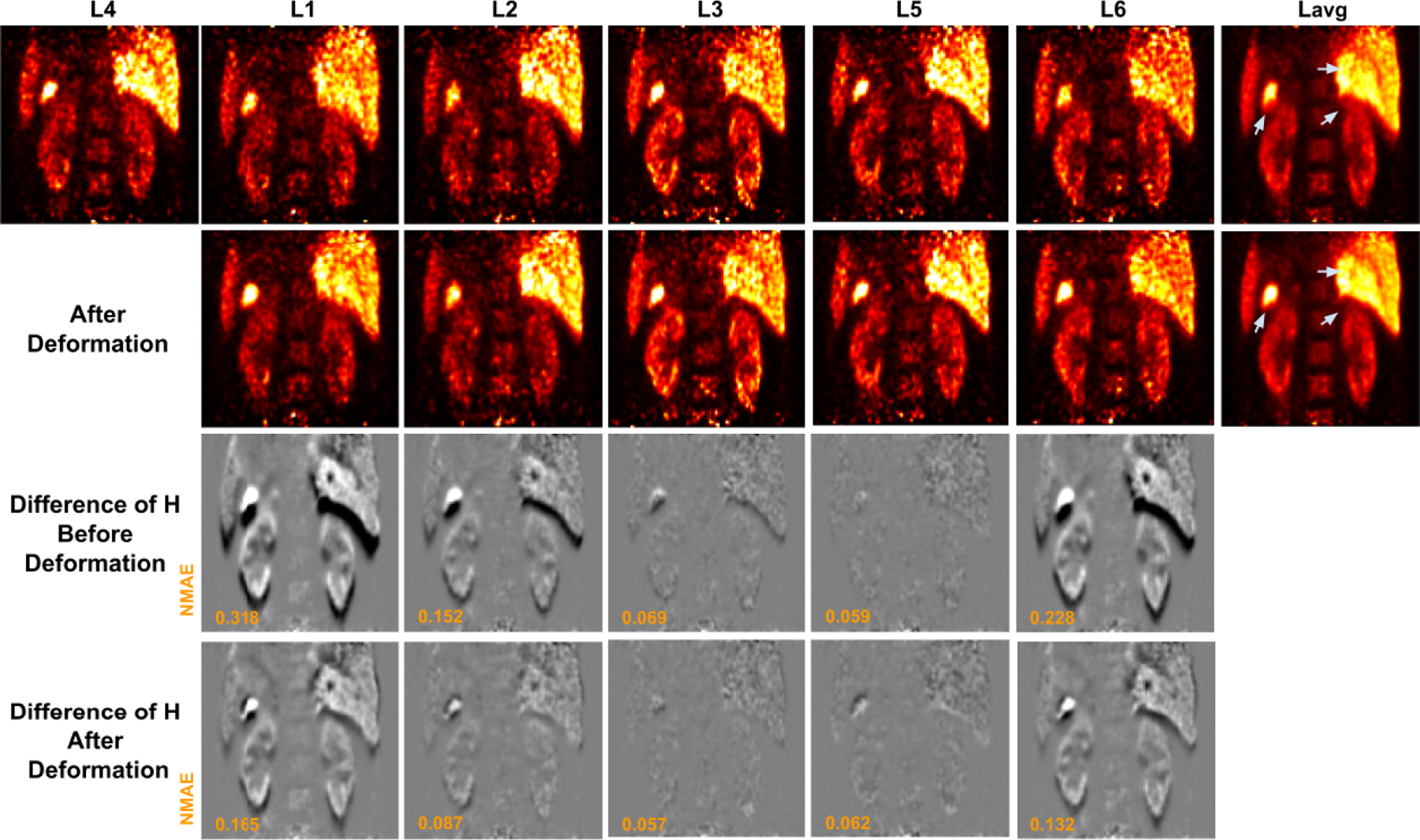
Low-dose gated image before and after deformation by our MDPET. The unregistered low-dose gated images *L_n_* and the corresponding averaged image *L_avg_* are shown in the 1st row. The deformed low-dose gated image *L_n_* and the corresponding averaged image *L_avg_* are shown in the 2nd row. The predicted transformations are applied to the corresponding high-dose gated images *H_n_*, where the difference of H between reference gate and source gate are visualized. The difference of *H* before and after registration over all gates are shown in the 3rd and 4th row, respectively. The motion blurred regions are indicated by gray arrows.

A sample set of low-dose gated PET images with and without applying the deformation fields predicted by our MDPET network is shown in Figure 4. The corresponding averaged images are provided as well. To assist the evaluation, difference images between the reference gate and each source gate with and without applying the transformation fields were calculated using the corresponding high-dose gated images. As we can see from the first row of Figure 4, the low-dose gated images with only 0.25% count level are noisy. Although directly averaging the low-dose gated images reduced the noise, important anatomical structure or pathological findings were blurred. As shown in the second row of Figure 4, our MDPET can accurately predict and deform each low-dose gated image to the reference low-dose gated image (L4), leading to sharper anatomic boundaries in the averaged image. Moreover, without applying the predicted deformation fields, significant amounts of misalignment can be observed between the reference gate and Gate 1 / Gate 6 / Gate 2 due to the position difference between expiration and inspiration motion (Figure 4, third row). The bright and dark intensity difference at the top and bottom of the kidney and liver indicated the error caused by the inter-gate motion. On the other hand, the position difference between the reference gate and Gate 3 / Gate 5 was small because the expiration phase is relatively long and steady. After applying the MDPET-predicted transformation fields, as illustrated in the fourth row, the differences in *H* were significantly reduced for the gates with large position difference. Specifically, the bright and dark errors at the top and bottom of the kidney and liver were reduced. The remaining differences were largely due to the different amount of intra-gate motion, which is larger for inspiration gates, i.e. Gated 1 / Gate 6 / Gate 2 in our experiments.

**Fig. 5.**
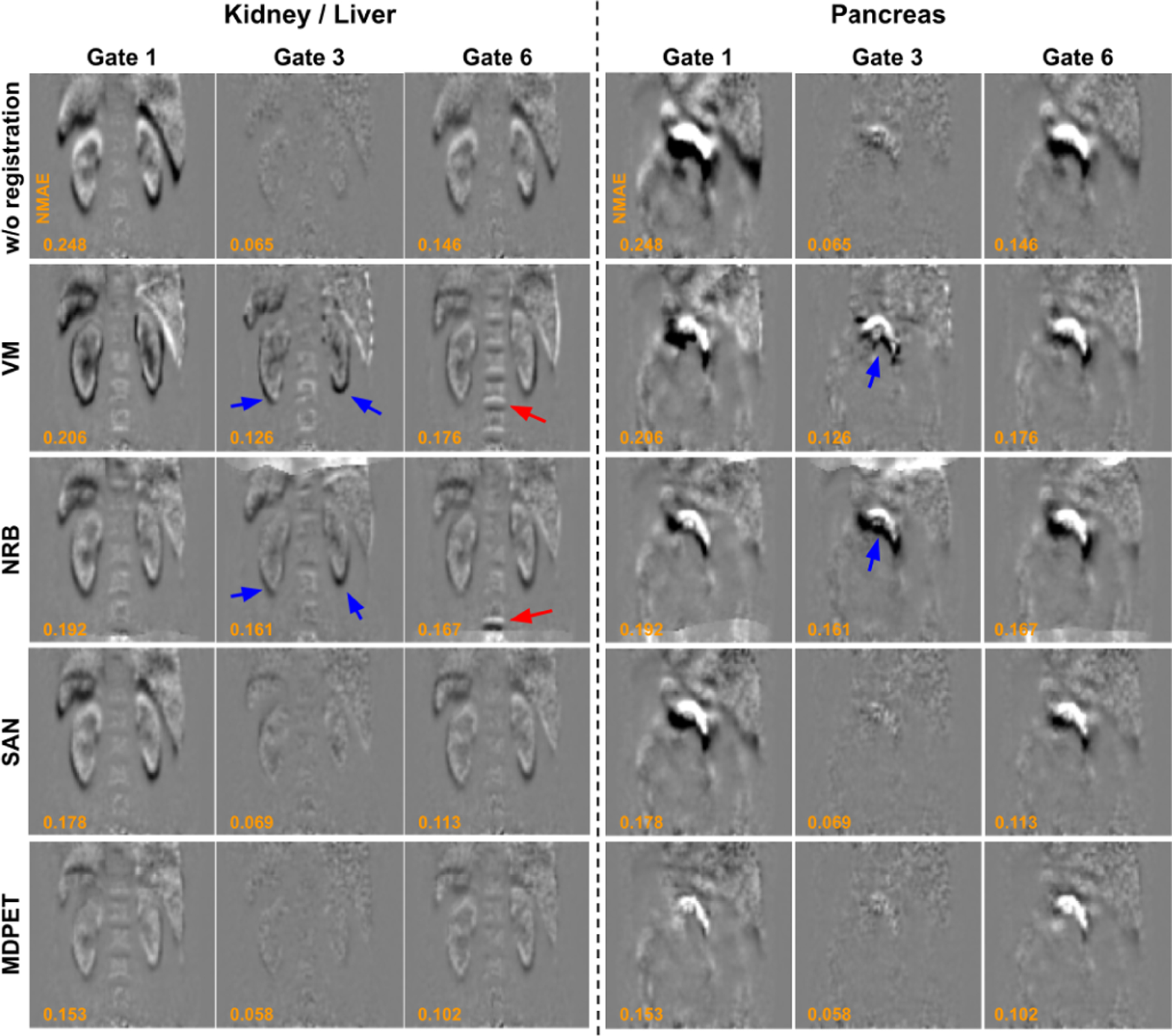
Comparison of registration errors between previous registration methods and our MDPET over Gate 1, Gate 3, and Gate 6 at kidney, liver, and pancreas regions. From top to bottom: without registration, VM [9], NRB [40], SAN [37], and our MDPET. Using NRB and VM, misalignment errors can be found in spine region at gate 6 (red arrows), and additional misalignment errors are introduced in kidney, liver and pancreas regions at Gate 3 (blue arrows).

The results of the proposed MDPET were compared with those of VM [9], NRB [40], and SAN [37]. Similar to the third and fourth rows of Figure 4, we used the difference image between *H* with and without applying the deformation to visualize the motion estimation errors (Figure 5). Two coronal slices containing different organs of interest are provided to assist the visual comparison. As we can see from the results for Gate 1 and Gate 6 in which large motion displacement was observed, even though VM and NRB were able to reduce the position difference in the kidney, liver and pancreas, they introduced additional misalignments in the spine regions that should remain unmoved over the scan. From the results of Gate 3 with minimal motion displacement, VM and NRB introduced additional misalignments. On the other hand, our previously proposed method, SAN, was able to better align the kidney, liver, and pancreas with less misalignments in the spine region for Gates 1, 3 and 6. The MDPET network further reduced the small residual misalignment errors in SAN for all the gates, providing superior motion estimation results as compared to other methods (Figure 5, bottom row).

The quantitative results are summarized in Table I. Similar to the assessment in Figure 5, we used the transformation field *T*_*n*_ estimated from low-dose gated images *L*_*n*_ to transform the corresponding high-dose gated images *H*_*n*_ to minimize the impact of noise on motion vector evaluation, and calculated the NMAE between the reference high-dose gated image *H*_*ref*_ and the transformed high-dose gated images ${\bar{H}}_{n}$. For Gate 1 and Gate 6 with large intra-gate motion, our MDPET was able to significantly reduce the NMAE from 0.185 to 0.110 for Gate 1 and from 0.136 to 0.091 for Gate 6, demonstrating superior motion estimation performance than SAN, VM and NRB. For gates with small or no intra-gate motion, such as Gate 3, our MDPET could maintain the overall alignment and finely adjust the small misalignment in local regions. Thus, we observed small NMAE reduction for Gate 2, Gate 3 and Gate 5 when using our MDPET. In contrast, NRB and VM both led to degradation of NMAE for Gate 2, Gate 3, and Gate 5. The results were even worse than those without applying motion estimation. For example, NRB increased NMAE from 0.065 to 0.121 at gate 3. Previous methods of VM and NRB are limited for accurate registration in the low-dose gated images, and our MDPET can generate reasonable registration across all the gates. The run time analysis is summarized in the last two columns of Table I. NRB with iterative optimization required the longest run time, about 1489 seconds on average using CPU. On the other hand, deep learning based VM and SAN could directly infer the transformation once the models are trained, thus requiring much shorter run time on CPU or GPU. Unlike VM and SAN that required 5 times of inference over different gates, our MDPET used all the gated images at once, thus further reducing the GPU run time to 0.54 seconds on average.

We also performed ablation study on motion estimation for our MDPET. The results are summarized in Table II. As we can see, the BiConvLSTM in our TSP-Net could improve the motion estimation performance. The performance was slightly further boosted by the additional adversarial learning. However, adding BiConvLSTM slightly increased the GPU run time from 0.38 seconds to 0.54 seconds.

### B. Denoising Different Motion-Compensated Images

**Fig. 6.**
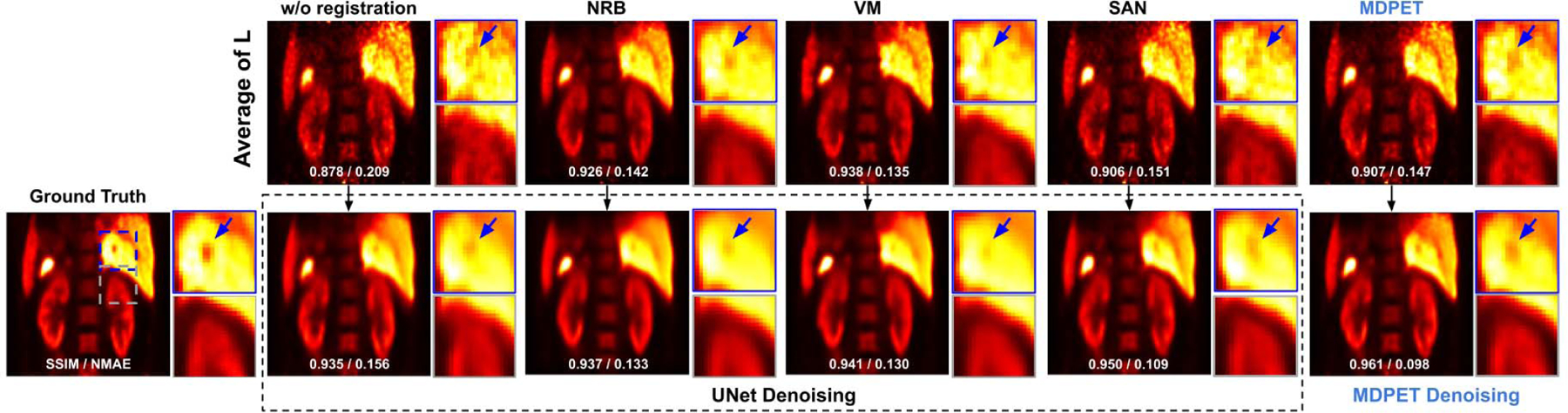
Comparison of denoising results. The averaged low-dose gated image generated from different motion estimation methods are shown in the 1st row. The corresponding denoised images are shown in the 2rd row. From left to right: ground truth, UNet denoising from the averaged image without any deformation, UNet denoising on the averaged image based on NRB-derived deformation fields, UNet denoising on the averaged image based on VM-derived deformation fields, UNet denoising on the averaged image based on SAN-derived deformation fields, and the end-to-end output from our MDPET. Our MDPET can reduce the motion blurring between the liver and kidney (gray box), as well as improving the visualization of small anatomic structures, such as portal veins (blue arrows).

After motion prediction, the averaged image of the transformed low-dose gated images was inputted into the denoising network to further reduce the noise. In Figure 6, we compared our MDPET results with other two-stage processing methods, including UNet denoising on the averaged image based on the NRB-derived transformation fields (NRB+UNet), UNet denoising on the averaged image based on the VM-predicted transformation fields (VM+UNet), and UNet denoising on the averaged image based on the SAN-predicted transformation fields (SAN+UNet). In NRB+UNet, the UNet was independently trained with paired motion-compensated averaged images from NRB and the ground truth high-dose image. The same UNet training protocol was used in VM+UNet and SAN+UNet. As observed in the figure, NRB+UNet and VM+UNet could reduce the global noise level. Subtle anatomic details, such as liver veins, were hard to observe for these two methods given the signal could have already been blurred out by motion in the input averaged image. On the other hand, in addition to reducing the global noise level, both SAN+UNet and our MDPET can better preserve anatomical details in the final image by efficiently reducing the motion blurring in the input averaged image. Our MDPET can generate anatomic details that best match with the ground-truth in terms of shape and intensity.

**Fig. 7.**
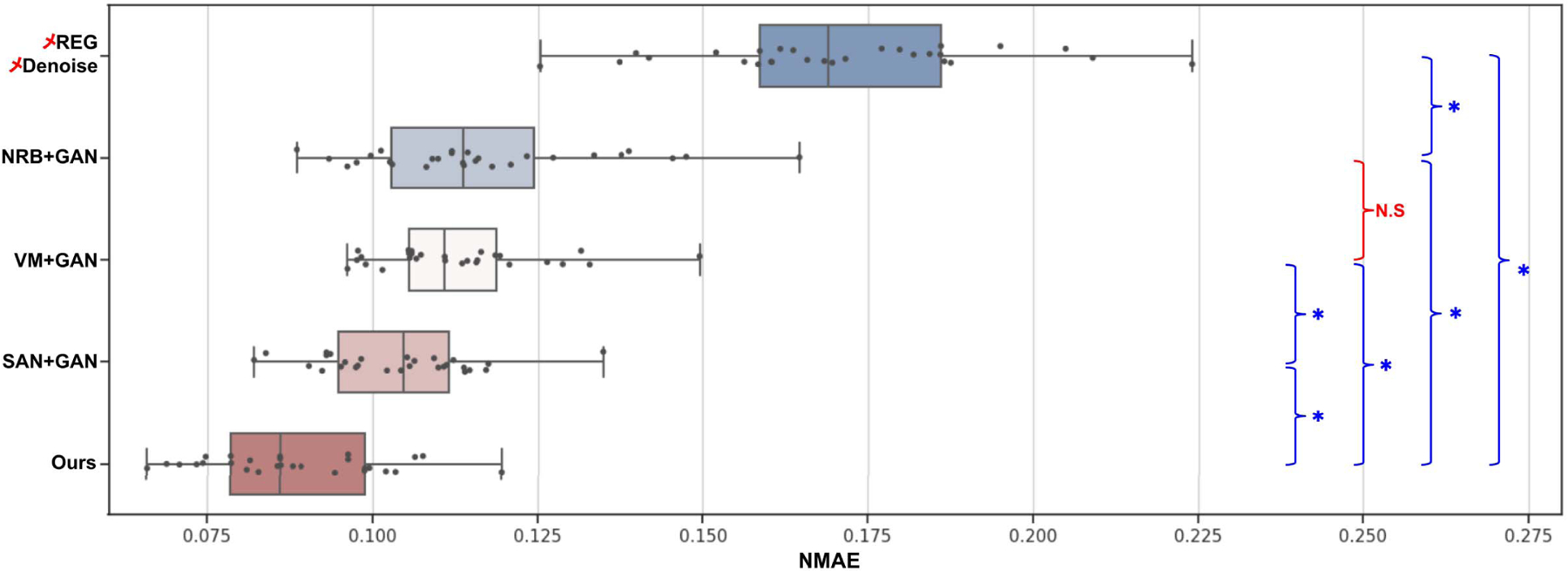
The boxplot results of all denoising testing images, where “*” means the difference are significant at *p* < 0.05, while “N.S” means not significant.

The quantitative results are summarized in Table III. In addition to UNet, we also explored the application of GAN with the same UNet generator in the two-stage methods, since adversarial learning is also implemented in our MDPET. Therefore, the quantitative results of our MDPET were compared not only with those of NRB+UNet / VM+UNet / SAN+UNet, but also with those of NRB+GAN / VM+GAN / SAN+GAN. As we can see, the evaluated image quality metrics were slightly improved while applying any of the two-stage processing methods, regardless of the incorporated image denoising network. The two-stage processing methods can reduce the NMAE from 0.17 to about 0.08. However, in the two-stage processing methods, changing the denoising network from UNet to GAN does not lead to significant improvements. On the other hand, our MDPET unifying motion estimation and denoising demonstrated the superior performance with mean NMAE=0.088, SSIM=0.966, and PSNR=32.28. Note that the image quality metrics for our MEPET’s averaged image (✓Ours+✗DN) were worse than those for NRB’s averaged image (✓NRB+✗DN) and VM’s averaged image (✓VM+✗DN). However, the denoising results based on our MDPET’s averaged image demonstrated the best performance. This is caused by the fact that NRB and VM register the image merely based on the image appearance, including anatomical structure and noise. Registering the noise will result in smoother averaged image, thus generating better image quality metrics for NRB and VM. Our MDPET registration can mitigate the impact from noise, thus providing averaged image with better anatomic details for denoising. The boxplot of our comparison results along with statistical analysis are summarized in Figure 7.

**Fig. 8.**
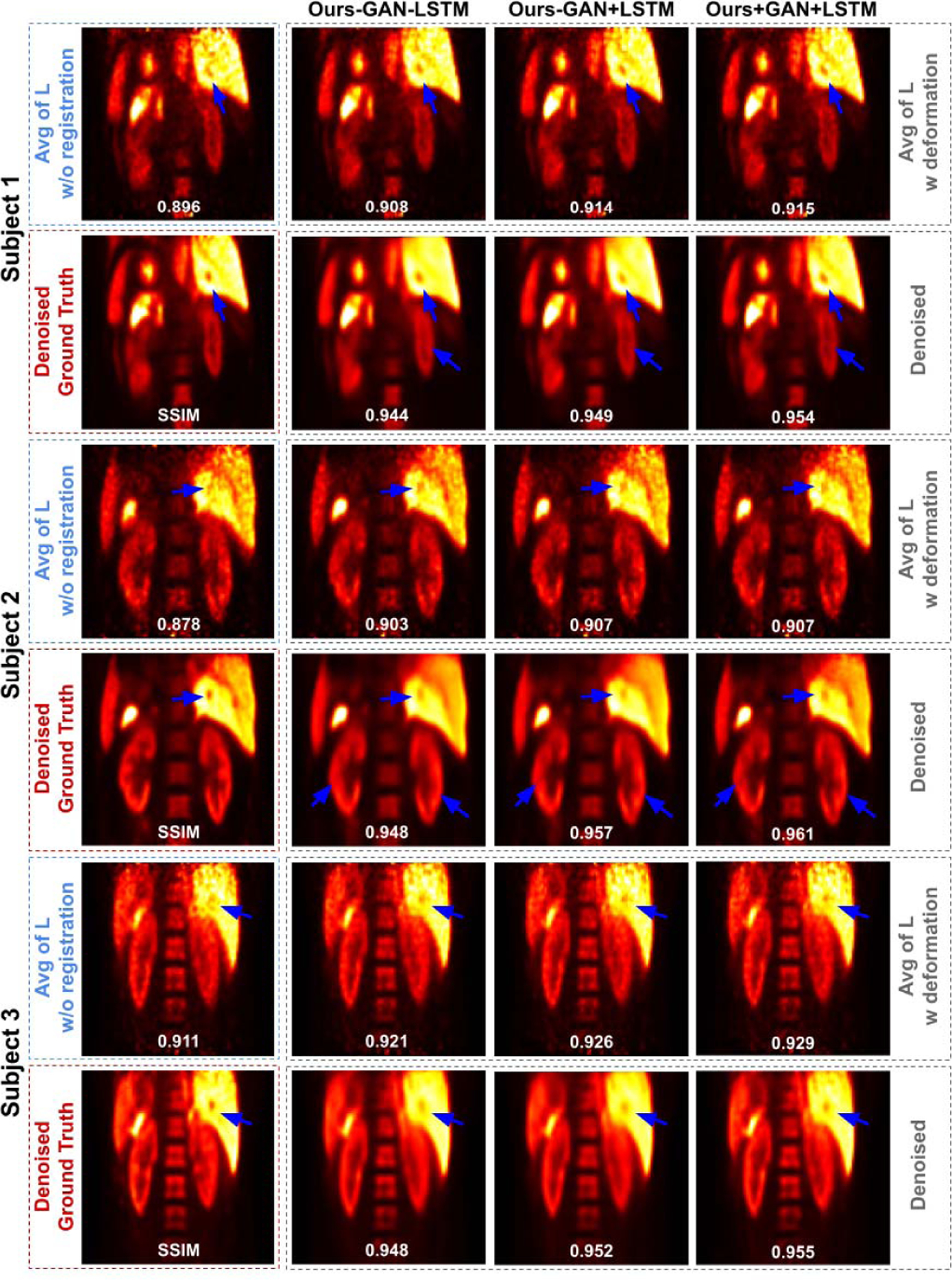
Three subjects with low-dose gated PET. The averaged images *L* and the corresponding denoised image from different MDPET configurations are shown in the 1st row and 2rd row in each patient’s image group. Motion blurred anatomic structure are recovered using our MDPET (blue arrows).

We also performed ablation study on denoising for our MDPET. The results are summarized in Table IV. According to Table II in the previous section, incorporating BiConvLSTM could improve the motion estimation performance thus generating sharper averaged image for denoising. Therefore, as we can observe from Table IV, adding BiConvLSTM could produce better image quality over the baseline MDPET. Moreover, adding adversarial learning could further improve the denoising performance. Three human subjects are illustrated in Figure 8. Overall, our MDPET with both BiConvLSTM and adversarial learning achieved the best motion estimation and denoising performance.

## V. Discussion and Conclusion

In this work, we proposed a unified motion estimation and denoising adversarial network, called MDPET, for generating motion-compensated low-noise PET image from low-dose respiratory gated PET. First, we developed a motion estimation module, TSP-Net, that can reliably estimate the motion from the low-dose gated images, which also incorporates the temporal motion features to improve the motion estimation. The basic unit of SP-Net in TSP-Net utilizes the denoised coarse-to-fine pyramid features to generate the motion features for each gate. Our TSP-Net then takes the motion features from each SP-Net into a recurrent layer to learn the temporal motion relationship over the gates, thus generating accurate motion estimation for all gates at once. Second, we unify the motion estimation network with a denoising network to directly generate motion-compensated low-noise PET images. Specifically, the gated images are deformed using the transformation fields predicted by TSP-Net and averaged such that all the counts in low-dose scan can be utilized to reduce the noise. Then, the averaged image is fed into a denoising network to further reduce the noise. A discriminator is added to the denoising output to enable adversarial learning for both motion estimation and denoising in our MDPET.

We demonstrated successful application on low-dose respiratory gated PET with evaluations on both motion estimation and denoising. For motion estimation, we compared with other previous motion estimation methods, including NRB, VM, and SAN. NRB and VM are not robust to noise in the low-dose gated images, thus leading to significant increases in registration errors in Gate 2 / Gate 3 / Gate 5, as illustrated in Table I. SAN with denoising first then motion estimation leads to better motion estimation as the noise in the low-dose gated images was first suppressed. However, SAN requires two-steps processing and requires 5 times inference for each study. On the other hand, our MDPET was able to generate superior motion estimation over all respiratory gates with the shortest inference time of 0.5 seconds. Ablation studies also demonstrated that adding the recurrent layer for temporal motion feature learning allows our MDPET to generate better motion estimation. For denoising, we compared our end-to-end denoising output with conventional two-stage processing methods, i.e. motion estimation then denoising. Because the motion estimation of NRB and VM are prone to error due to high noise level in the low-dose gated images, their averaged image may have already suffered from residual motion blurring and the denoising network cannot recover the motion blurred signals. The denoising results from SAN’s averaged images are more reasonable as SAN can better align the low-dose gated images. However, the motion estimation and final denoising are in two separate stages. The denoising network trained separately may not be able to correct the residual motion blurring in the averaged image. In this case, our MDPET is an end-to-end framework and the denoising output based on our motion-compensated averaged image provides the best reconstructed image quality with PSNR = 32.28.

The presented work also has potential limitations. First of all, the denoising result is still not as distinct as the ground truth from high-dose gated image. In our current MDPET implementation, we use UNet as our denoising network because its efficiency has been extensively studied and demonstrated in literature [15]. However, the denoising network in our MDPET is interchangeable with other advanced denoising networks [13], [17], [46], [47] to potentially further improve the image quality. Moreover, perceptual loss [17] could also be incorporated into the MDPET to help further recover the image details. However, perceptual loss is currently only available for 2D image but not 3D imaging data as in our work. In addition, more patient data could be collected for training our MDPET in the future for further improving the performance. Secondly, our work only addressed the inter-gate motion (motion between gates) but not the intra-gate motion (motion within each gate) for low-dose gated PET. The gated images may already suffer from intra-gate motion blurring, potentially affecting our inter-gate motion estimation and the subsequent denoising. Although we have chosen the end-expiration gate image with the least intra-gate motion as the ground truth for supervising the MDPET’s output to mitigate the impact, future work could also consider event-by-event listmode based correction to further limit the amount of intra-gate motion in each gate. Finally, current image reconstructions were based OSEM with 1 iteration. Additional iteration numbers and filtering settings need to be investigated in our future work.

Our MDPET also suggests several potential clinical applications for our future studies. First of all, since MDPET could generate high-quality motion compensated PET image under low-dose injection protocol, our generated image is potentially useful for diagnosis purposes, especially for abdominal regions where respiratory motion is inevitable. Second, our MDPET is also potentially useful for registering continuous bed motion (CBM) multi-pass for whole body dynamic PET. To elaborate, each CBM pass is scanned with a short time period (2–5 min) that contains a high noise level, similar to low-dose gated PET. The respiratory motion is inevitable in a CBM acquisition. Thus, our method can potentially apply to CBM inter-pass and intra-pass motion correction. Lastly, our method could potentially be adapted to deviceless low-dose gating reconstruction as well.

In conclusion, we proposed a unified motion estimation and denoising adversarial network for low-dose gated PET. The experimental results using human data show that our MDPET can accurately estimate the motion over the noisy low-dose gated images and simultaneously produce high-quality motion-compensated denoised PET image. Future work would also investigate the potential of further improving the performance of MDPET by substituting our current MDPET framework with different state-of-the-art motion estimation and denoising sub-networks on different applications.

## Figures and Tables

**TABLE I:** Comparison of Different Motion Estimation Methods for Different Gates. NMAE Is Calculated Based on the *H* Transformed by the Predicted *T*. The Run Time of Each Algorithm on CPU and GPU Is Shown on the Last Two Columns.

NMAE	Gate 1	Gate 2	Gate 3	Gate 4	Gate 5	Gate 6	GPU sec	CPU sec
w/o registration	0.1846	0.1066	0.0653	-	0.0659	0.1360	0	0
NRB [40]	0.1564↓*	0.1347↑*	0.1212↑*	-	0.1288↑*	0.1339↓^†^	-	1489
VM [9]	0.1362↓*	0.1202↑*	0.1126↑*	-	0.1144↑*	0.1232↓*	2.1	220
SAN [37]	0.1298↓*	0.0882↓*	0.0682↑^†^	-	0.0751↑*	0.1103↓*	4.3	423
Ours	0.1098↓*	0.0749↓*	0.0582↓*	-	0.0619↓*	0.0908↓*	0.54	59

↓ and ↑ Mean the NMAE Decrease and Increase as Compared to Baseline NMAE Without Registration, Respectively.

“*” Means the Difference to the Baseline NMAE Without Registration Are Significant at *p* < 0.05, While “†” Means Not Significant

**TABLE II:** Ablation Study on Our MDPET in Terms of Motion Estimation. ± LSTM Means MDPET With or Without BiConvLSTM and ± GAN Means MDPET With or Without Adversarial Learning.

NMAE	Gate 1	Gate 2	Gate 3	Gate 4	Gate 5	Gate 6	GPU sec	CPU sec
Ours-LSTM-GAN	0.1283	0.0849	0.0660	-	0.0701	0.1010	0.38	36
Ours+LSTM-GAN	0.1116*	0.0762*	0.0588*	-	0.0623*	0.0920*	0.54	59
Ours+LSTM+GAN	0.1098*	0.0749*	0.0582*	-	0.0619*	0.0908*	0.54	59

“*” Means the Difference to the Baseline (1st Row) Are Significant at *p <* 0.05

**TABLE III:** Comparison of Denoising Performance on Different Motion-Compensated Images. Our MDPET Is Compared With 1) SAN and 2) Two-Stage Processing Methods That Consist of Motion Estimation and Denoising (DN).

Method-mean(std)	NMAE	SSIM	PSNR
✗REG+✗DN	.1712(.0225)	.9018(.0175)	25.87(1.87)
✓NRB+✗DN	.1174(.0198)	.9424(.0096)	28.97(1.79)
NRB+UNet	.1166(.0177)	.9479(.0068)	29.49(1.85)
NRB+GAN	.1147(.0179)	.9489(.0071)	29.66(1.91)
✓VM+✗DN	.1165(.0130)	.9431(.0080)	28.98(1.90)
VM+UNet	.1125(.0124)	.9480(.0052)	29.43(1.99)
VM+GAN	.1128(.0130)	.9490(.0061)	29.48(1.98)
✓SAN+✗DN	.1401(.0187)	.9191(.0154)	27.99(1.49)
SAN+UNet	.1062(.0122)	.9498(.0061)	30.31(1.87)
SAN+GAN	.1036(.0117)	.9503(.0061)	30.87(1.79)
✓Ours+✗DN	.1383(.0185)	.9193(.0153)	28.14(1.46)
Ours	.0883(.0133)	.9669(.0054)	32.28(1.89)

✓ and ✗ Denote Use or Not Use of a Specific Processing Stage. For Example, ✓NRB+✗DN Means NRB Is Used For Estimating the Motion and Generating The Averaged Image, but No Denoising Step Is Applied. The Corresponding Boxplot Comparison Results With Statistical Analysis Are Shown in Figure 7

**TABLE IV:** Ablation Study on Our MDPET in Terms of Denoising. ± LSTM Means MDPET With or Without BiConvLSTM and ± GAN Means MDPET With or Without Adversarial Learning.

Method-mean(std)	NMAE	SSIM	PSNR
Ours-LSTM-GAN	.1058(.0140)	.9587(.0063)	30.95(1.89)
Ours+LSTM-GAN	.0921(.0137)	.9613(.0062)	31.64(1.88)
Ours+LSTM+GAN	.0883(.0133)*	.9669(.0054)*	32.28(1.89)*

“*” Means the Difference to the Baseline (1st Row) Are Significant at *p <* 0.05
